# AMPA-Type Glutamate Receptors Associated With Vascular Smooth Muscle Cell Subpopulations in Atherosclerosis and Vascular Injury

**DOI:** 10.3389/fcvm.2021.655869

**Published:** 2021-04-20

**Authors:** Alessandro L. Gallina, Urszula Rykaczewska, Robert C. Wirka, April S. Caravaca, Vladimir S. Shavva, Mohamad Youness, Glykeria Karadimou, Mariette Lengquist, Anton Razuvaev, Gabrielle Paulsson-Berne, Thomas Quertermous, Anton Gisterå, Stephen G. Malin, Laura Tarnawski, Ljubica Matic, Peder S. Olofsson

**Affiliations:** ^1^Laboratory of Immunobiology, Center for Bioelectronic Medicine, Department of Medicine, Center for Molecular Medicine, Karolinska Institute, Stockholm, Sweden; ^2^Vascular Surgery, Department of Molecular Medicine and Surgery, Karolinska Institute, Stockholm, Sweden; ^3^Division of Cardiology, University of North Carolina School of Medicine, Chapel Hill, NC, United States; ^4^Department of Cell Biology and Physiology, University of North Carolina, Chapel Hill, NC, United States; ^5^McAllister Heart Institute, University of North Carolina, Chapel Hill, NC, United States; ^6^Department of Cardiovascular Sciences, Katholieke Universiteit Leuven, Leuven, Belgium; ^7^Division of Cardiovascular Medicine and Cardiovascular Institute, School of Medicine, Stanford University, California, CA, United States; ^8^Institute of Bioelectronic Medicine, Feinstein Institutes for Medical Research, Manhasset, NY, United States

**Keywords:** inflammation, neuroscience, neurotransmitter, GluA1, GluA2, smooth muscle cells, atherosclerosis, glutamate

## Abstract

**Objectives and Aims:** Vascular smooth muscle cells (VSMCs) are key constituents of both normal arteries and atherosclerotic plaques. They have an ability to adapt to changes in the local environment by undergoing phenotypic modulation. An improved understanding of the mechanisms that regulate VSMC phenotypic changes may provide insights that suggest new therapeutic targets in treatment of cardiovascular disease (CVD). The amino-acid glutamate has been associated with CVD risk and VSMCs metabolism in experimental models, and glutamate receptors regulate VSMC biology and promote pulmonary vascular remodeling. However, glutamate-signaling in human atherosclerosis has not been explored.

**Methods and Results:** We identified glutamate receptors and glutamate metabolism-related enzymes in VSMCs from human atherosclerotic lesions, as determined by single cell RNA sequencing and microarray analysis. Expression of the receptor subunits glutamate receptor, ionotropic, α-amino-3-hydroxy-5-methyl-4-isoxazolepropionic (AMPA)-type subunit 1 (GRIA1) and 2 (GRIA2) was restricted to cells of mesenchymal origin, primarily VSMCs, as confirmed by immunostaining. In a rat model of arterial injury and repair, changes of GRIA1 and GRIA2 mRNA level were most pronounced at time points associated with VSMC proliferation, migration, and phenotypic modulation. *In vitro*, human carotid artery SMCs expressed GRIA1, and selective AMPA-type receptor blocking inhibited expression of typical contractile markers and promoted pathways associated with VSMC phenotypic modulation. In our biobank of human carotid endarterectomies, low expression of AMPA-type receptor subunits was associated with higher content of inflammatory cells and a higher frequency of adverse clinical events such as stroke.

**Conclusion:** AMPA-type glutamate receptors are expressed in VSMCs and are associated with phenotypic modulation. Patients suffering from adverse clinical events showed significantly lower mRNA level of GRIA1 and GRIA2 in their atherosclerotic lesions compared to asymptomatic patients. These results warrant further mapping of neurotransmitter signaling in the pathogenesis of human atherosclerosis.

**Graphical Abstract:**
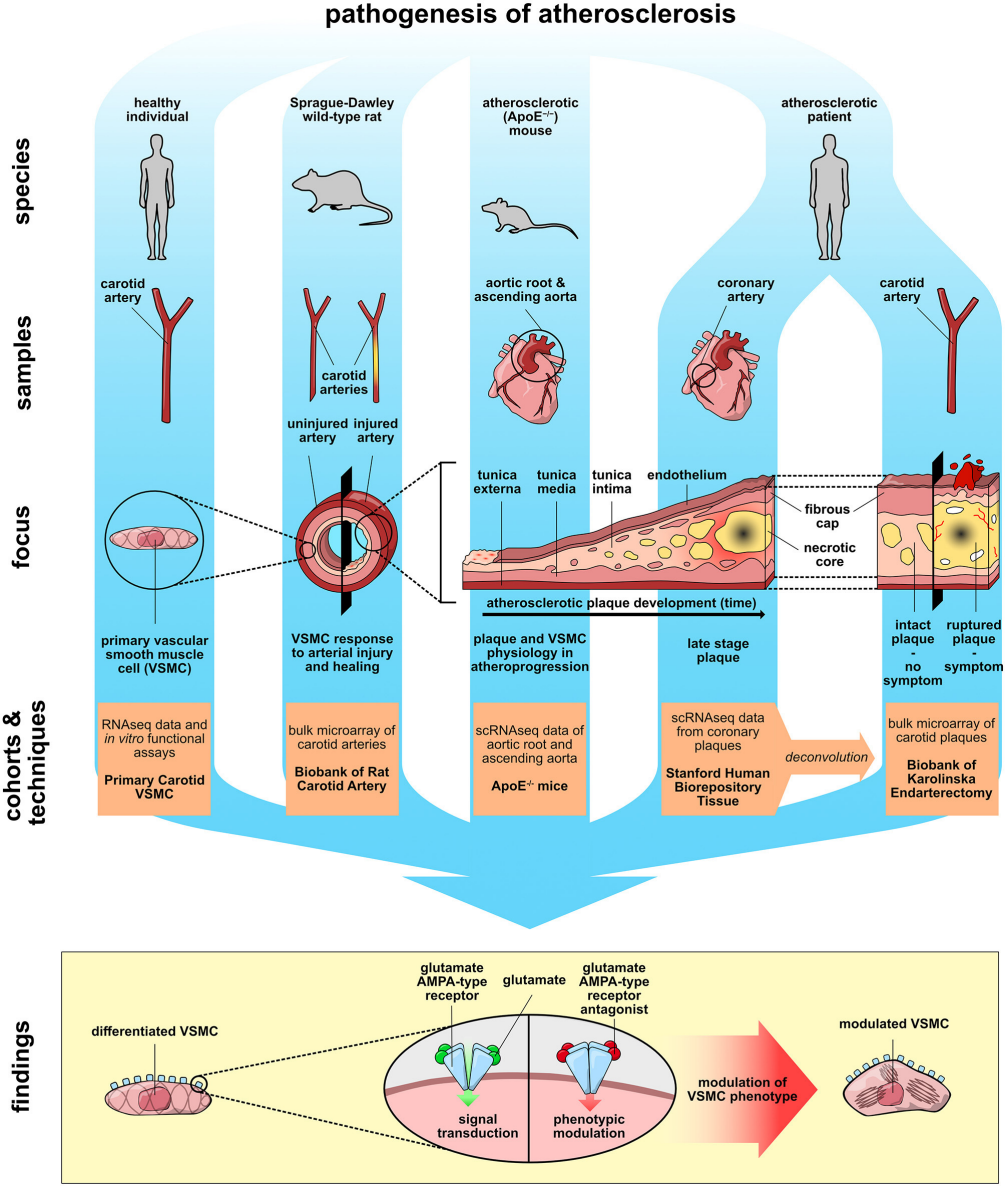
Aspects of atherosclerosis pathogenesis were studied in cell culture, rodent models of vascular injury and atherosclerosis, and human arterial biopsies. Together, the findings reveal that AMPA-type glutamate receptors on vascular smooth muscle cells regulate genes associated with cellular phenotype.

## Introduction

Cardiovascular disease (CVD) accompanied by chronic inflammation causes disability, suffering, and death, and in 2017, accounted for 45% of deaths in Europe (1). Available treatments fail to completely prevent atherosclerosis development, which is a major cause of cardiovascular events and death. Vascular smooth muscle cells (VSMCs) are a key constituent of normal arteries and participate in the structural and functional changes in arteries that underlie atheroprogression. They have intrinsic plasticity and an ability to phenotypically modulate (2–4). For example, VSMCs are abundantly present in the fibrous cap that often demarcates advanced atherosclerotic plaques (3). Reduced stability of the fibrous cap increases the risk of atherothrombotic clinical events, including heart attacks and stroke (3, 5). Recent lineage-tracing studies support that VSMCs are not terminally differentiated and represent a larger proportion of cells than previously appreciated as they undergo various forms of trans-differentiation in response to local environmental stimuli, in both arterial remodeling and atherosclerotic plaques (6–8). Numerous studies highlight the significance of the VSMC phenotype for plaque stability (7, 8). While VSMC proliferation commonly appears to support vascular repair in atherosclerosis, specific VSMC phenotypes may instead promote inflammation and plaque vulnerability (2, 3). Accordingly, improved understanding of plaque VSMC phenotype and its regulation in atherosclerosis is important (2).

Recent observations have identified a possible role for metabolic shifts in VSMCs in the pathogenesis of vascular inflammation, arterial remodeling, and atheroprogression (2, 5, 7, 8). In particular, observations on the role of glutamate, a non-essential amino acid, are interesting in this context. Glutamate, an important source of energy and a key signaling molecule, is present in plasma and synthesized from the precursor glutamine, which is the most abundant free amino acid (9, 10). It signals through receptors that are abundantly expressed in the central nervous system and several other tissues (11, 12), including the ionotropic receptors α-amino-3-hydroxy-5-methyl-4-isoxazolepropionic acid (AMPA)-, N-methyl-D-aspartate (NMDA)-, kainate (KA)-, and delta-types. These receptors are found in the heart, skin, bone, and other organs, but their function in these tissues has not been fully elucidated (12, 13). Interestingly, in a mouse model of vascular remodeling, NMDA-receptor deficiency attenuates VSMC proliferation, and blocking glutamate NMDA-receptor in a rat model of pulmonary hypertension reduces vascular remodeling (14). Other observations suggest that changes in glutamate metabolism play a role in the pathophysiology of CVD. For example, a human variant of a key enzyme for glutamate turnover, the glutamate-ammonia ligase (*GLUL*) gene, appears to contribute to coronary heart disease risk by affecting glutamate/glutamine metabolism (15). Moreover, glutamate blood levels are associated with a higher CVD risk (16).

In light of the experimental data on glutamate receptor biology in the pathogenesis of vascular remodeling in pulmonary hypertension and the observed associations between changes in glutamate signaling and severity of CVD, we proceeded to investigate glutamate receptors in end-stage human atherosclerosis, and in experimental vascular injury.

## Materials and Methods

### The Biobank of Karolinska Endarterectomies (BiKE) Cohort

Patients undergoing surgery for symptomatic or asymptomatic, high-grade (>50% NASCET) (17) carotid stenosis at the Department of Vascular Surgery, Karolinska University Hospital, Stockholm, Sweden, were consecutively enrolled in the study and clinical data were recorded on admission. Patients without qualifying symptoms within 6 months prior to surgery were categorized as asymptomatic (AS) and the indication for carotid endarterectomy (CEA) were based on results from the Asymptomatic Carotid Surgery Trial (ACST) (18). Patients from the symptomatic group were further stratified by symptoms of plaque instability, defined as transitory ischemic attack (TIA) and minor stroke (MS) as more severe symptoms, and *amaurosis fugax* (AF) as a less severe symptom. Carotid endarterectomies (carotid plaques, CP) were collected at surgery and retained within the Biobank of Karolinska Endarterectomies (BiKE). The BiKE study cohort demographics, details of sample collection, processing and analyses were previously described (19–21). The studies are approved by the Ethical Committee of Stockholm and follow the guidelines of the Declaration of Helsinki. Studies are performed with the following ethical permit numbers: BiKE EPN DNr 95-276/277; 01-199; 02-146; 02-147; 04-225/4; 04-97 5T; 2005/83-31; 2007/281-31/4; 2009/4:2; 2009/9-31/4; 2009/295- 31/2; 2009/512-31/2; 2009/2000-32; 2010/1022-31/1; 2010/730-31/2; 2011/196-31/1; 2011/629-32; 2011/950-32; 2012/619-32; 2012/916-31/4; 2012/1096-31/2; 2012/1279-32; 2013/615-31/4; 2012/2188-31-5; 2013/2048-32; 2013/2137-32; 2015/1338-32; 2015/2108-31/5; 2017/508-32; and 2018/954-32. All human samples and data in BiKE are collected with written informed consent from patients or organ donors' guardians. Tissue and blood sampling are conducted as part of the ordinary medical and surgical procedures.

### scRNAseq Data of Human Coronary Artery Plaques

Coronary arteries were dissected from explanted hearts of transplant recipients who provided their written consent prior to the procedure and their inclusion in this cohort. Tissues were obtained from the Human Biorepository Tissue Research Bank under the Department of Cardiothoracic Surgery, with approval from the Stanford University Institutional Review Board. The basic clinical characteristics of the patients included (*n* = 4) in the study were previously described (22). The top 100 differently expressed gene markers were used to distinguish each cluster (reference cluster) from the remaining clusters in the human scRNAseq dataset (*n* = 4 patients) (22).

### Microarray Data Deconvolution

Deconvolution was performed using previously described methodology (23) via the Cibersort web software (https://cibersort.stanford.edu). In brief, cell populations were defined using pre-assigned “cell-type signature markers” (22) and used to estimate the relative frequencies of cell populations in the BiKE biobank microarray data. This gene-by-cell type matrix file obtained by scRNAseq contained expression levels for the top 26 significant genes for each of the 15 major cell types.

### Atherosclerosis Prone Mouse Model and scRNAseq

The genotype of SMC lineage-tracing (SMC^lin^) mice, used for scRNAseq, was: *Tg*^*Myh*11−*CreERT*2^, *ROSA*^*tdT*/+^, and *ApoE*^−/−^. In brief, immediately after euthanasia, the aortic root and ascending aorta were excised, up to the level of the brachiocephalic artery. Tissue was mechanically and enzymatically dissociated and the cell suspension was passed through a strainer. Multiple mice were used at each time point and single cell suspensions obtained. For the SMC^lin^ genotype, three mice were used at baseline, and three mice were used at both 8 and 16 weeks of disease. Details of sample collection, processing and analyses were as previously described (22). The animal study protocol was approved by the Administrative Panel on Laboratory Animal Care at Stanford University.

### Rat Carotid Artery Balloon Injury

Rat carotid artery injury and healing response model was studied by global mRNA expression profiling *via* microarrays, as described previously (24). Carotid artery balloon injury was performed on 69 male Sprague-Dawley rats as previously described (24). Isoflurane inhalation (IsoFlo®Vet, Abbott Laboratories Ltd, Berkshire, England) was used for anesthesia. Briefly, the left carotid artery was isolated, an arteriotomy was performed on the external carotid artery and the common carotid artery was de-endothelialized 3 times with a 2F Fogarty catheter. Animals were euthanized with 5% isoflurane overdose by inhalation (maintaining isoflurane exposure until 1 min after breathing stops) at several time points: directly after injury (0 h), 2, 20 h, 2, 5 days, 2, 6, and 12 weeks following vascular injury. Both the left (injured) and contralateral, right (uninjured) common carotid arteries were collected for analyses. Briefly, microarray transcript analysis was performed with Affymetrix GeneTitan Rat Gene ST v1.1 arrays. Robust multi-array average normalization and batch effect correction of the microarray dataset were performed. Final analyses were performed using GraphPad Prism 6 and a two-sided Student's *t-*test and Bonferroni correction for multiple comparisons. All animal studies were performed with a local Committee approval (Ethical Board of North Stockholm, Dnr N181/16; N137/14) and conform to the guidelines from Directive 2010/63/EU of the European Parliament on the protection of animals used for scientific purposes.

### Immunohistochemistry (IHC)

In brief, 5 μm sections were deparaffinized in HistoLab Clear (Histolab) and rehydrated in graded ethanol. For antigen retrieval, slides were subjected to high-pressure boiling in Diva Decloaker buffer pH 6.0 (BioCare Medical). The slides were stained with Hematoxylin QS (Vector Laboratories), dehydrated and mounted in Pertex (Histolab). Images were acquired using an automated SlideScanner system (Hamamatsu Nanozoomer 2.0 HT).

### Immunofluorescence (IF)

Consecutive 5 μm sections of human carotid plaques from the BiKE cohort were fixed in 4% PFA and embedded in paraffin. Slides were deparaffinized in HistoLab Clear (Histolab) and rehydrated in graded ethanol. For antigen retrieval, slides were subjected to high-pressure boiling in Diva Decloaker buffer pH 6.0 (BioCare Medical). Human carotid artery SMCs were prepared for staining by culturing on glass coverslips coated with fibronectin (Invitrogen), washing with cold PBS and fixation with 4% PFA for 10 min. Thereafter, blocking was performed with 3% bovine serum albumin (BSA) in 0.05% TBS Tween. Primary antibodies used include: polyclonal rabbit anti-human GRIA1 (Cat. No. Ab31232; 1:200; Abcam), monoclonal rabbit anti-human GRIA2 (Cat. No. Ab133477; 1:1,500; Abcam), monoclonal mouse anti-human smooth muscle α-actin (Cat. No. M0851; 1:400; Dako), monoclonal mouse anti-human CD68 (Cat. No. M0876; Dako), and monoclonal mouse anti-human von Willebrand factor (Cat. No. M0616; Dako), diluted in blocking buffer. Secondary antibodies used were anti-mouse and anti-rabbit DyLight 488 and biotin conjugated, respectively (Vector Laboratories). Streptavidin conjugated with DyLight 594 (Vector Laboratories) was used for the detection of anti-rabbit biotin conjugated antibody. Nuclei were stained with DAPI and slides were mounted with Dako mounting media (Agilent). Consecutive slides from the same plaque were stained with isotype controls for GRIA1 (Rabbit polyclonal IgG; Cat. No. ab171870; Abcam) and GRIA2 (rabbit monoclonal IgG; Cat. No. ab172730; Abcam), and used as negative controls (Supplementary Figure 1A).

Passage 6 primary human carotid artery SMCs (Cat. No. 3514-05a; Cell Applications) were cultured on glass coverslips coated with fibronectin (Cat. No. PHE0023, Invitrogen) in 5% CO_2_ humidified incubator at 37°C, in complete Human Smooth Muscle Cell Growth Medium (Cat. No. CC-3181; Lonza) with addition of supplements and growth factors (Cat. No. CC-4149; Lonza). Cells were serum starved for 24 h, and fixed with 4% PFA. They were stained with a monoclonal mouse anti-pan-AMPA receptor (Cat. No. MABN832; Merck Millipore). Anti-mouse biotin conjugated (Vector Laboratories) was used as secondary antibody. Streptavidin conjugated with DyLight 488 (Vector Laboratories) was used for detection. The cytoskeleton was visualized using Phalloidin iFluor 594 (Cat. No. ab176757; Abcam). Nuclei were stained with DAPI and slides were mounted with Dako mounting media (Agilent). Negative control staining was performed on fixed cells incubated with “Polymer negative control serum” for mouse and rabbit antibodies (Cat. No. nc499h; Biorad) (Supplementary Figure 1B).

Images were acquired with a Nikon Eclipse T12 confocal microscope and processed with Fiji open source software.

### Primary Human Carotid Artery Smooth Muscle Cell (hcSMC) Culture

Primary hcSMCs (Cat. No. 3514-05a; Cell Applications) of passage 5–7 were cultured in 5% CO_2_ humidified incubator at 37°C, in complete Human Smooth Muscle Cell Growth Medium (Cat. No. CC-3181; Lonza) with addition of supplements and growth factors (Cat. No. CC-4149; Lonza). Phenotypic characterization of these cells with respect to the expression of contractile markers was performed previously (25). 2,3-dioxo-6-nitro-7-sulfamoyl-benzo[f]quinoxaline disodium salt (NBQX) (Cat. No. 0373; Tocris) was used as selective AMPA/KA ionotropic glutamate receptor antagonist (26). NBQX was freshly dissolved in PBS for stock solution aliquots at 5 mM. NBQX aliquots were used in supplemented growth media as described above, with a final concentration of 25 μM. Cells were serum starved for 24 h, then the media was replaced with NBQX-supplemented media for the next 24 h. Supernatant was collected for ELISA analysis and hcSMCs were rinsed with cold PBS and then lysed in RLT lysis buffer (Cat No. 79216; Qiagen) for downstream analyses.

### BrdU Incorporation Assay

hcSMCs (Cat. No. 3514-05a; Cell Applications) were plated on 96-well plates coated with fibronectin (Cat. No. PHE0023, Invitrogen) and left in 5% CO_2_ humidified incubator at 37°C to adhere. Cell proliferation was assessed using the colorimetric immunoassay measuring BrdU incorporation during DNA synthesis (#11647229001, Roche), in accordance to the manufacturers' protocol. Eleven replicates were analyzed for each condition.

### Click-IT TUNEL Assay

Incorporation of dUPTs-labeling at the ends of DNA fragments was assessed accordingly to the manufacturer's protocol (#C10245, Thermo Fisher). Briefly, hcSMCs (Cat. No. 3514-05a; Cell Applications) were plated on 24-well plates coated with fibronectin (Cat. No. PHE0023, Invitrogen) and left in 5% CO_2_ humidified incubator at 37°C to adhere. Cells were washed with PBS and then fixed with 4% paraformaldehyde (10 min, RT) and subsequently permeabilized for 10 min using 0.05% Tween in PBS. After washing, cells were incubated with TdT reaction cocktail for 60 min at 37°C, followed by the addition of Click-iT reaction cocktail for 30 min. DAPI was used to stain nuclei. Mounting was performed with DAKO fluorescent mounting medium.

### Semi-quantitative Real-Time PCR (qPCR)

RNA was isolated using RNeasy kit (at No. 74106; Qiagen) with DNase treatment, according to the manufacturers' protocol. cDNA was generated using the High-Capacity RNA-to-cDNA Kit (Cat. No. 4387406; ThermoFisher). qPCR reactions were performed mixing pre-diluted cDNA with the 20x TaqMan Gene Expression Assays (Cat. No. 4331182; Applied Biosystems) and TaqMan Universal PCR Master Mix (2x) (Cat. No. 4305719; Applied Biosystems). The qPCR was run using 7900 HT real-time PCR system (Applied Biosystems). All samples were analyzed in duplicate. Results were normalized to *PPIA*. FAM dye-labeled probes (TaqMan) used: *GRIA2* (Cat. No. Hs00181348-m1); *GRIA1* (Cat. No. Hs00181331-m1); *PPIA* (Cat. No. Hs99999904-m1).

### Cytokine Analysis

Supernatant from hcSMC was collected after 24 h of culture in different defined conditions, diluted 1:15 in PBS, and analyzed for IL-6 concentration by ELISA (R&D Systems) performed according to manufacturer instructions. Plates were read using a VersaMax Microplate Reader (Molecular Devices).

### RNA-Sequencing

hcSMCs at passage 7 were washed with cold PBS and then lysed in RLT lysis buffer (Qiagen). RNA was isolated using RNeasy kit (Qiagen), according to the manufacturers' protocol. RNA integrity was evaluated with an Agilent 2100 Bioanalyzer (Agilent Technologies). The RNA integrity numbers exceeded 8.5 in all samples. RNA was selected using Poly(A) RNA Selection Kit (Lexogen) and sequencing libraries prepared with Lexogen QuantSeq V2. DNA fragments of 200–800 bp for RNA-seq were selected. Cluster generation and sequencing was carried out by using the Illumina HiSeq 2500 system with a read length of 50 nucleotides (single-read). Sequence reads that passed the Illumina quality filtering were aligned to the human reference genome GRCh38 with star-aligner and gene annotation from ENSEMBL (GRCh38.95). Analysis of differential expression of mRNA was using the DeSeq2 software at default settings. This dataset was submitted to the European Nucleotide Archive (ENA) (https://www.ebi.ac.uk/ena/browser/home) with the accession reference ERP127239.

### Bioinformatic and Statistical Analyses

The BiKE microarray dataset is available from Gene Expression Omnibus (GSE21545). Robust multi-array average normalization and batch effect correction of the microarray data were performed and processed log_2_ gene expression data were returned. Analyses were performed with GraphPad Prism 8 using a two-tailed Student's *t*-test and correction for multiple comparisons according to the Bonferroni-Dunn method. Fold change values were calculated as “log_2_(average mRNA expression in the experimental group/average mRNA expression in the reference group).” Gene set enrichment analyses on Gene Ontology (GO) terms in BiKE cohort were performed with GeneMania (www.genemania.org) and GOrilla (http://cbl-gorilla.cs.technion.ac.il) software. Filtering of overlapping GO categories was performed using Revigo software (http://revigo.irb.hr). Pearson correlations were calculated to determine the association between mRNA expression levels from microarrays. Large-scale global correlation analyses were produced using Morpheus software (http://software.broadinstitute.org/morpheus).

In RNAseq data, to calculate transcript length accounting for the presence of multiple splice isoforms, lengths of all unique exons from all transcripts of a given gene were added together. For overlapping exons, only a unique part of the repeating exon was added to the overall transcript length. Exon data was retrieved from Ensembl database using R/Bioconductor package biomaRt (27, 28). Gene set enrichment analyses on Gene Ontology (GO) terms in RNAseq data from hcSMCs were performed by using R/Bioconductor package topGO (29). To test for enrichment, Kolmogorov-Smirnov Testing was used. Additional data about the genes was extracted with GeneCards (www.genecards.org) and PubMed. To visualize changes in genes of the MAPK signaling pathway R/Bioconductor packages KEGGREST (30) and pathview (31) were used. Heatmaps were created using R/Bioconductor package pheatmap (32). In all analyses *p* < 0.05 was considered significant.

## Results

### AMPA-Type Glutamate Receptors Are Expressed in Human Atherosclerotic Plaques

**Figure 1:**
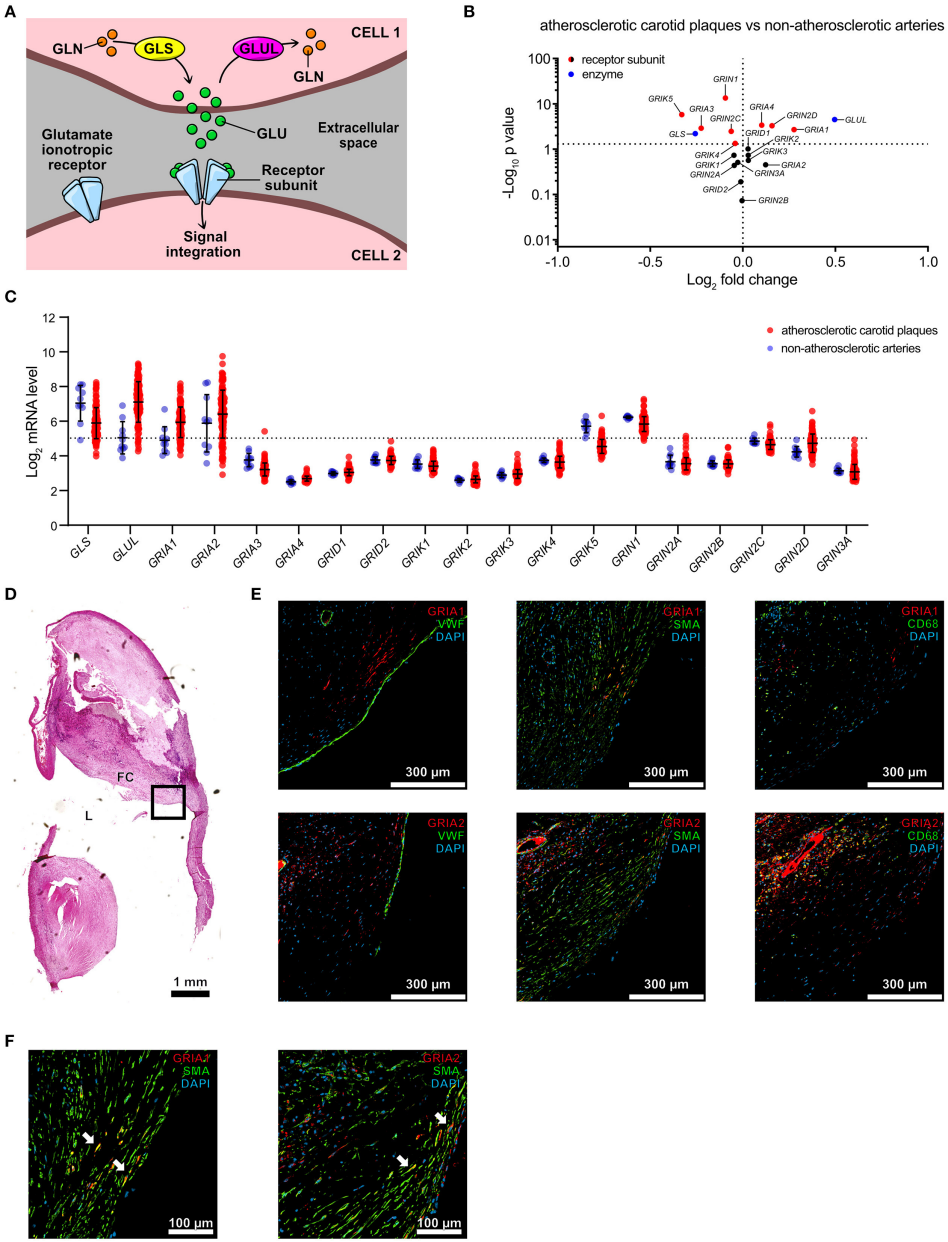
AMPA-type glutamate receptors are expressed in human atherosclerotic plaques. **(A)** Schematic representation of cell-to-cell glutamatergic communication mechanisms mediated by glutamate ionotropic receptor receptors (Gln, Glutamine; Glu, glutamate; GLS, glutaminase; GLUL, glutamine synthetase). **(B)** Volcano plot visualization of differentially expressed genes between non-atherosclerotic reference arteries vs. carotid atherosclerotic plaques in the BiKE cohort. Fold change is expressed as “log_2_ (mean expression in carotid atherosclerotic plaques/mean expression in non-atherosclerotic reference arteries).” Differences between groups were analyzed using unpaired Student's *t*-test. Glutamate metabolism-related genes are shown in blue, glutamate ionotropic receptors subunits are shown in red when statistically differently expressed, in black when not statistically significantly expressed. *GLS*, Glutaminase; *GLUL*, Glutamate-ammonia ligase; *GRIA1-4*, Glutamate receptor, ionotropic, AMPA 1-4; *GRID1-2*, Glutamate receptor, ionotropic, delta 1-2; *GRIK1-5*, Glutamate receptor, ionotropic, kainate 1-5; *GRIN1, 2A–D, 3A*, Glutamate receptor, ionotropic, N-methyl D-aspartate 1, 2A-D, 3A. Missing genes from these families correspond to missing probes in the microarray dataset. **(C)** Dot plot showing mRNA levels of glutamate turnover-related genes and glutamate ionotropic receptors subunits, described in **(B)**, in atherosclerotic carotid plaques (*n* = 127, red) and non-atherosclerotic control arteries (*n* = 10, blue) from the BiKE cohort microarray data. Dots represent log_2_ mRNA levels. Middle bar indicates the median mRNA expression and error bars represent SD. **(D)** Section from a human carotid plaque from the BiKE cohort stained with Hematoxylin QS. L, lumen; FC, Fibrous cap. The black square indicates the region of interest for immune-fluorescent staining shown in the following panels. **(E)** Consecutive histological sections of the human carotid plaque shown in **(D)** were stained with antibodies against GRIA1, GRIA2, von Willebrand factor (VWF), smooth muscle actin (SMA), and CD68. Fibrous cap regions are shown. Nuclei visualized with DAPI (blue). **(F)** Higher magnification of consecutive histological sections of a representative human carotid plaque shown in **(E)** stained with antibodies against GRIA1, SMA, and GRIA2. White arrows indicate co-localization of SMA (green) with GRIA1 or GRIA2 (red).

To investigate the presence of mediators associated with glutamate signaling and metabolism (Figure 1A) in human atherosclerotic plaques, we queried the microarray data from our Biobank of Carotid Endarterectomies (BiKE) (19, 20) for mRNA expression of enzymes associated with glutamate turnover and components of ionotropic glutamate receptors of NMDA-, AMPA-, KA- and delta-type. The glutamate metabolism-related enzyme Glutaminase (*GLS*) and Glutamate-ammonia ligase (*GLUL*) transcripts were detected in biopsies from both non-atherosclerotic reference arteries (*n* = 10) and atherosclerotic lesions (*n* = 127) (Figure 1B, Supplementary Table 1). *GLS* was significantly higher in non-atherosclerotic arteries compared to carotid atherosclerotic plaques, while *GLUL* was significantly lower (Figure 1B, Supplementary Table 1). Among the highly expressed genes, there was no significant difference in NMDA- and delta-type receptor transcript levels between non-atherosclerotic arteries and carotid plaques with the exception of Glutamate receptor, ionotropic, N-methyl D-aspartate 1 (*GRIN1*), which was more abundant in non-atherosclerotic biopsies than in atherosclerotic lesions (Figures 1B,C, Supplementary Table 1). KA-type receptor transcript levels were low, with the exception of Glutamate receptor, ionotropic, kainate 5 (*GRIK5*), which was more abundant in non-atherosclerotic biopsies than in atherosclerotic lesions (Figures 1B,C, Supplementary Table 1). Interestingly, transcripts of the receptor subunits Glutamate receptor, ionotropic, AMPA-type subunit 1 and 2 (*GRIA1* and *GRIA2*) were found in atherosclerotic lesions and *GRIA1* levels were significantly higher in atherosclerotic lesions than in non-atherosclerotic arteries (Figures 1B,C, Supplementary Table 1). Double-immunofluorescence stainings of consecutive slides from a representative carotid atherosclerotic plaque biopsy from the BiKE biobank (19, 20) (plaque morphology shown in Figure 1D) were performed for GRIA1 or GRIA2 proteins and markers of cell types including von Willebrand factor (VWF), smooth muscle actin (SMA), and CD68 (Figure 1E). Staining showed co-localization of SMA and GRIA1 or GRIA2 proteins in plaques' fibrous cap (Figure 1F), suggesting a VSMC-restricted expression of these AMPA-type glutamate receptors. These observations indicate that the machinery for glutamate turnover and signaling (Figure 1A) is present in human atherosclerotic lesions.

### VSMCs Express AMPA-Type Glutamate Receptors in Atherosclerotic Plaques

**Figure 2:**
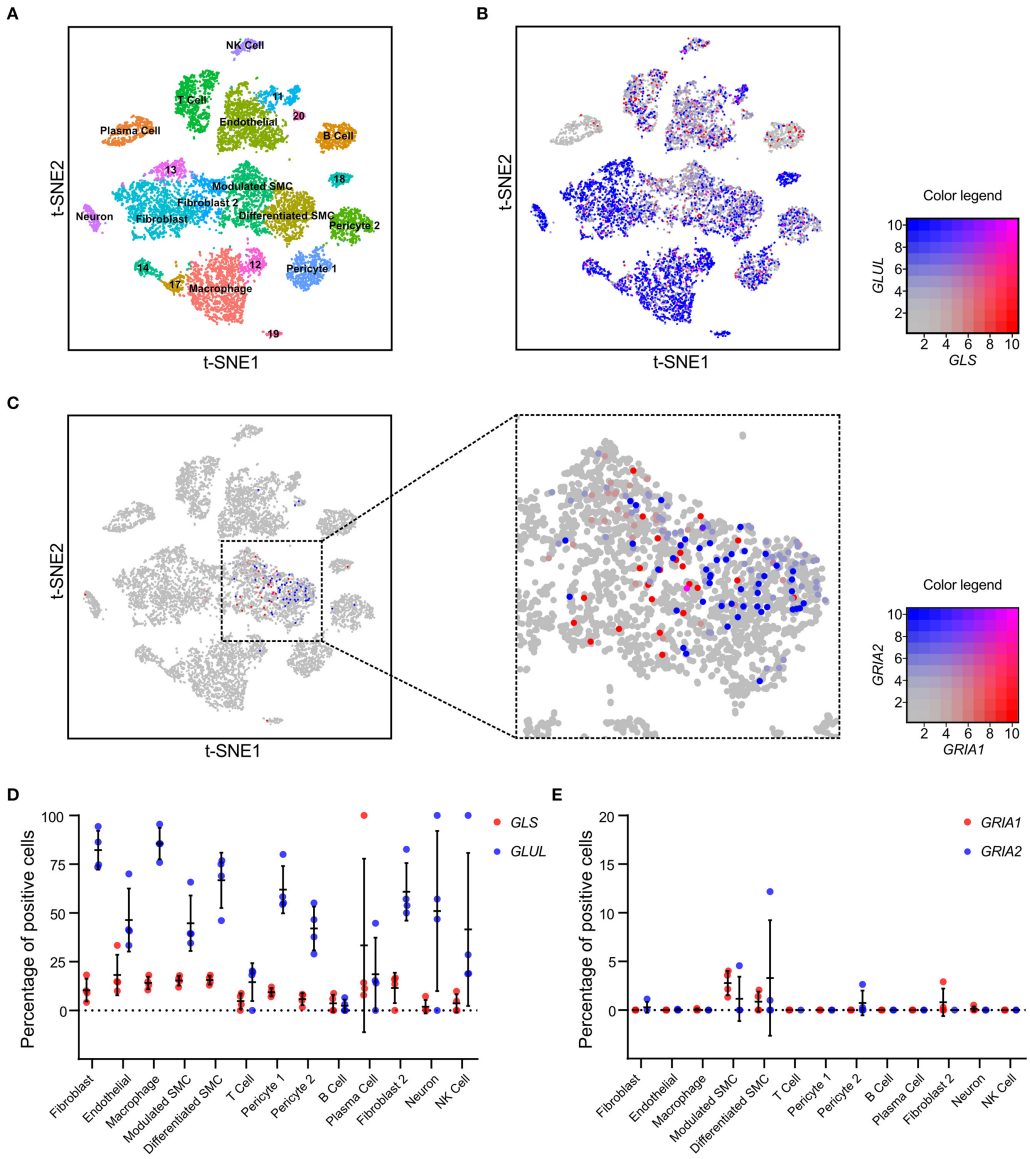
VSMCs express AMPA-type glutamate receptors in atherosclerotic plaques. **(A)** tSNE visualization of single cell transcriptomic analysis of plaques (*n* = 5) derived from the right coronary artery of human patients (*n* = 4), colored according to broad cell clustering as indicated in the figure. Numbers denote unidentified clusters. NK, natural killer; SMC, smooth muscle cell. **(B)** tSNE visualization of single cell transcriptomic analysis of *GLS* (red) and *GLUL* (blue) expression overlaid on the cell clusters from **(A)**. Color legend (right) indicating relative expression levels for *GLS1* on x-axis and *GLUL* on y-axis. **(C)** tSNE visualization of single cell transcriptomic analysis of *GRIA1* (red) and *GRIA2* (blue) expression overlaid on the cell clusters from **(A)**. Color legend (right) indicating relative expression levels for *GRIA1* on x-axis and *GRIA2* on y-axis. **(D)** Dot plot visualization of the percentage of cells positive for *GLS* (red) and *GLUL* (blue) expression within the identified cell clusters. Error bars indicate SD, *n* = 4 patients. **(E)** Dot plot visualization of the percentage of cells positive for *GRIA1* (red) and *GRIA2* (blue) expression within the identified cell clusters. Error bars indicate SD, *n* = 4 patients.

The cellular distribution of glutamate signaling related transcripts was further analyzed in human atherosclerotic coronary plaques (*n* = 5, from 4 patients) using single-cell RNA sequencing (scRNAseq) (22) (Figure 2A, Supplementary Table 2). Key enzymes and receptors for glutamate metabolism, and signaling, were found in coronary atherosclerotic plaques, where 56% ± 14 of all analyzed cells were positive for *GLUL*, and 12% ± 3.1 were positive for *GLS*. *GLUL* was primarily found in fibroblasts, macrophages, differentiated VSMCs, and pericytes, while *GLS* was predominantly detected in endothelial cells, fibroblasts, differentiated VSMCs, modulated VSMCs, and plasma cells (Figures 2B,D, Supplementary Table 2). Expression of the AMPA-type glutamate receptor subunits *GRIA1* and *GRIA*2 was only detected in cell populations of mesenchymal origin, with the overwhelming majority identified as VSMCs (Figures 2C,E, Supplementary Table 2). Publicly available scRNAseq data from atherosclerotic lesions of ApoE^−/−^ mice (*n* = 3 per timepoint, collection at baseline, 8, and 16 weeks after initiation of high fat diet, 2 experiments) were analyzed for expression of *Glul, Gls, Gria1* and *Gria2* (22). Glul, Gls, and Gria1-expressing VSMCs were observed at a similar frequency as in human atherosclerotic lesions. *Gria2* was not detected (Supplementary Figure 2).

### AMPA-Type Receptors *Gria1* and *Gria2* Associated With VSMC Phenotypic Switch in Arterial Remodeling and Repair

**Figure 3:**
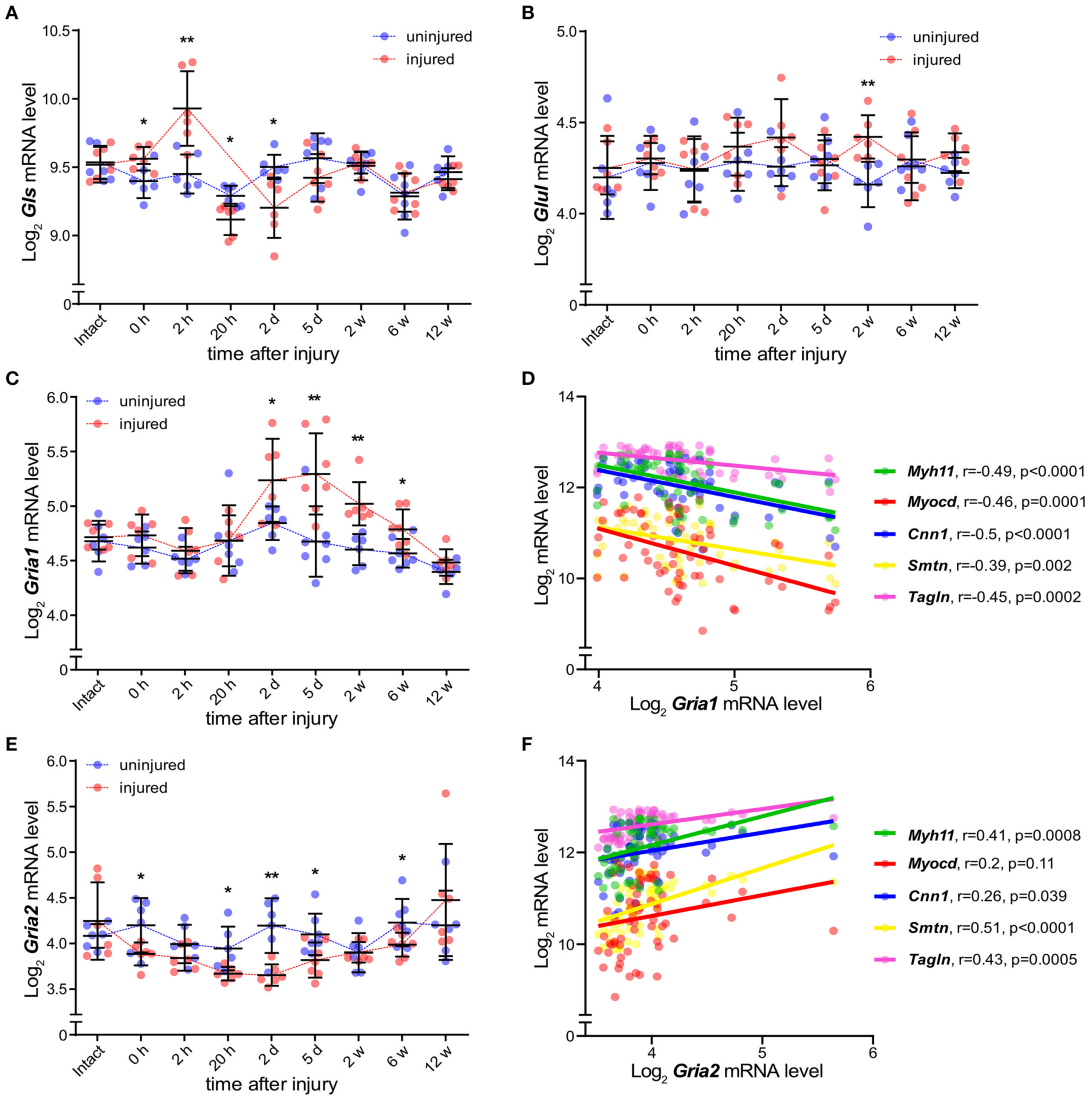
AMPA-type receptors *Gria1* and *Gria2* associate with SMC phenotypic switch in arterial repair. **(A,B)** Dot plots showing mRNA levels of *Gls* **(A)** and *Glul* **(B)** during the course of carotid artery injury and healing in injured (red) and uninjured (blue) rat carotid arteries (*n* = 6–7 per time point) over the course of 12 weeks. **(C)** mRNA levels of *Gria1* during the course of rat carotid artery injury and healing response described in **(A,B)**. **(D)** mRNA levels of *Gria1* plotted versus typical markers of smooth muscle cells in injured arteries from all time points (*n* = 64) (r_P_ = Pearson r). **(E)** mRNA levels of *Gria2* during the course of rat carotid artery injury and healing response described in **(A,B)**. **(F)** mRNA levels of *Gria2* plotted versus typical markers of smooth muscle cells in injured arteries from all time points (*n* = 64) (r_P_ = Pearson r). mRNA levels were measured by microarray assay. Dots represent log_2_ mRNA levels. Middle bar indicates the median mRNA level and error bars represent SD. *p < 0.05, **p < 0.01, calculated using Kruskal-Wallis ANOVA test followed by Bonferroni-Dunn correction for multiple comparisons. Myosin heavy chain 11 (*Myh11*, green), Myocardin (*Myocd*, red), Calponin 1 (*Cnn1*, blue), Smoothelin (*Smtn*, yellow), and Transgelin (*Tagln*, purple).

Based on the scRNAseq results, we hypothesized that expression of *GRIA1* and *GRIA2* is associated with VSMC phenotypic shift. To investigate this, we measured carotid artery transcript expression of key elements associated with glutamate signaling in a model of rat arterial injury and repair over a period of 12 weeks (24). This model is well-established for studies of intimal hyperplasia and VSMC phenotypic modulation, and three distinct phases of arterial repair and VSMC switch are observed in this model at 0–2 h (early), 20 h−5 days (intermediate) and 2–12 weeks (late) (24). Transcripts associated with modulated VSMC were elevated during the intermediate phase (24). In this model, measurements in the injured carotid artery are compared with the contralateral, uninjured carotid artery at each time point. Biopsies from animals that underwent sham surgery were obtained from both carotid arteries and designated “intact arteries.” *Gls* and *Glul*, were expressed throughout the 12-week period in biopsies from intact, injured, and uninjured arteries (Figures 3A,B). From 2 days until 6 weeks after injury, *Gria1* mRNA levels were significantly higher in injured arteries compared with both contralateral uninjured arteries and “intact artery” biopsies (Figure 3C). *Gria1* mRNA levels were significantly negatively correlated with the established VSMC markers myocardin (*Myocd*), calponin 1 (*Cnn1*), smoothelin (*Smtn*), smooth muscle myosin heavy chain 11 (*Myh11*), and transgelin (*Tagln*) (Figure 3D). In contrast to *Gria1, Gria2* mRNA was significantly decreased in the injured arteries as compared with the contralateral uninjured arteries and intact arteries (Figure 3E). There was a significant positive correlation between mRNA levels of *Gria2* and *Cnn1, Smtn, Myh11*, and *Tagln* (Figure 3F). *Gria2* were significantly lower than *Gria1* mRNA levels throughout the injury and repair process (average Log_2_ difference = 0.45; Student's *t*-test *p* < 0.0001). We were unable to visualize AMPA-type receptor proteins in the rat samples.

### AMPA Receptors Regulate a Gene Set Associated With VSMC Phenotypic Modulation *in vitro*

**Figure 4:**
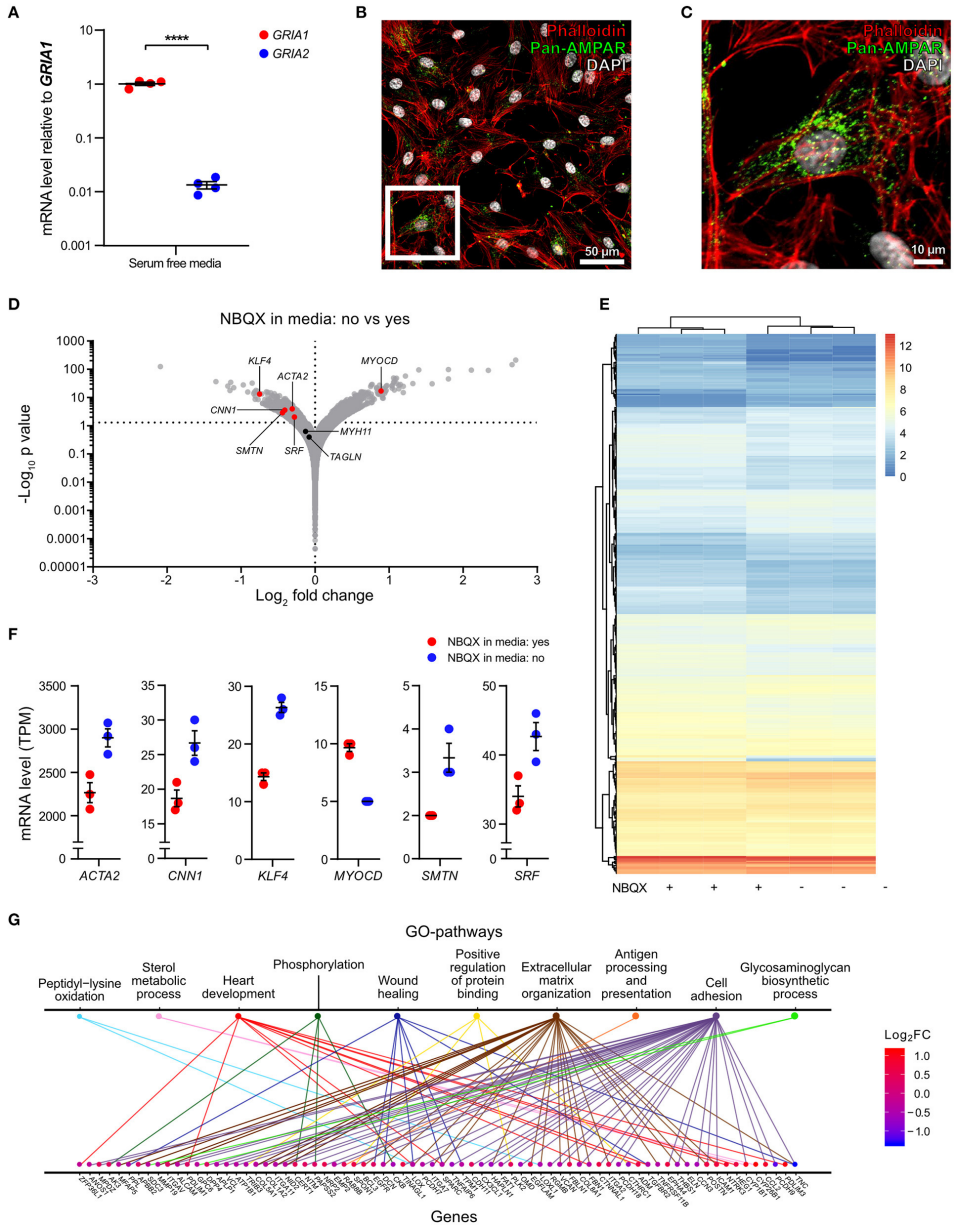
AMPA receptors regulate a gene set associated with VSMC phenotypic modulation *in vitro*. **(A)** Dot plot indicating the relative expression of *GRIA1*, and *GRIA2* in human carotid smooth muscle cells (hcSMCs) maintained for 24 h in serum free media (*n* = 4). mRNA levels were quantified using qPCR and are shown as relative levels to the average of *GRIA1* mRNA. Middle lines indicate mean with error bars indicating SEM. ****p < 0.0001 (unpaired Student's *t*-test). **(B,C)** hcSMCs maintained in serum free media for 24 h were stained using anti-pan-AMPA receptor (pan-AMPAR) antibody (green), phalloidin (red), and DAPI (blue), and fluorescence visualized using a Nikon confocal microscope. White square indicate the magnified area shown in **(C)**. **(D)** Volcano plot visualization of gene expression fold changes and *p-*values from RNAseq data. mRNA was isolated from hcSMCs grown in serum-free media in the presence (*n* = 3) or absence (*n* = 3) of the AMPA receptor antagonist 2,3-dihydroxy-6-nitro-7-sulfamoyl-benzo[f]quinoxaline disodium salt (NBQX) for 24 h. SMC markers are shown in red when their expression is statistically significantly different between conditions, otherwise markers are represented in black. Actin alpha 2 (*ACTA2*), Kruppel-like factor 4 (*KLF4*), Myosin heavy chain 11 (*MYH11*), Myocardin (*MYOCD*), Calponin 1 (*CNN1*), Smoothelin (*SMTN*), Serum response factor (*SRF*), and Transgelin (*TAGLN*). **(E)** Hierarchical clustering analysis and heatmap visualization of the transcript per million (TPM) counts > 2 of the significantly differently expressed genes from **(D)**. **(F)** Dot plots showing gene expression of significant differently expressed SMC markers from **(B)** in hcSMCs grown in serum free media in the presence (*n* = 3; blue) or absence (*n* = 3; red) of the AMPA receptor antagonist NBQX. Dots represent TPM. The middle bar indicates the median gene expression and error bars represent SEM. **(G)** Distribution of the significantly regulated genes in the top 10 enriched Gene ontology (GO) pathways. The genes are color coded according to their log_2_ fold change value: blue—reduced, red—increased in NBQX-treated hcSMCs.

There is a scarcity of information on AMPA receptor function in VSMCs. To address this, we cultured human carotid vascular SMCs (hcSMCs) in the presence or absence of the AMPA receptor antagonist 2,3-Dioxo-6-nitro-1,2,3,4-tetrahydrobenzo[f]quinoxalin-7-sulfonamide disodium salt (NBQX) (26). We found that hcSMCs cultured in serum free media express *GRIA1* and *GRIA2 in vitro*. mRNA levels of *GRIA1* were ~100-fold higher than *GRIA2* (Figure 4A) and AMPA-type glutamate receptor protein was detected by immunofluorescence in hcSMCs (Figures 4B,C). We next exposed hcSMCs to NBQX. BrdU incorporation assay showed lower proliferation in cultures exposed to the AMPA receptor antagonist NBQX as compared with vehicle (Supplementary Figure 3A). This exposure did not significantly affect apoptosis as measured by TUNEL staining (Supplementary Figure 3B). RNA sequencing (RNAseq) showed significant differences in transcript levels of 464 genes between cultures exposed to NBQX or vehicle, respectively (Figure 4D, Supplementary Table 3). Levels of the 464 significantly differentially expressed transcripts were analyzed by unsupervised hierarchical clustering and the two experimental groups clustered separately (Figure 4E). mRNA levels of the VSMC markers for contractile VSMCs (*ACTA2, CNN1, SRF, SMTN*) (4, 7, 19, 24) were lower in cultures exposed to NBQX compared with vehicle exposure, except the major transcription factor of the VSMC lineage *MYOCD*, levels of which were higher (Figures 4D,F). Of note, Interleukin 6 (*IL-6*) mRNA levels were significantly higher in hcSMCs exposed to NBQX (TPM = 160 ± 8.5) compared to hcSMCs exposed to vehicle (TPM = 107 ± 4.0) (Supplementary Figure 4A, Supplementary Table 3) and the difference in mRNA levels was reflected in culture supernatant IL-6 protein levels as measured by ELISA (1,500 pg/mL ± 21 vs. 2,280 pg/mL ± 101) (Supplementary Figure 4B). Moreover, IL-6 plasma levels in BiKE cohort patients correlated negatively with *GRIA1* plaque expression (r_P_ = −0.23; *p* = 0.015) (Supplementary Figure 4C). Gene ontology classification of the differently expressed genes revealed overrepresentation of genes involved in extracellular matrix organization, cell adhesion, and wound healing (Figure 4G, Supplementary Figure 5, Supplementary Table 4), suggesting that AMPA-type receptors regulate expression of a gene set involved in phenotypic modulation in hcSMCs. In addition, GO and KEGG pathway analysis showed that transcripts associated with MAPK and ERK signaling pathways were enriched in samples exposed to NBQX (Supplementary Table 5, Supplementary Figure 6).

### Low AMPA-Type Receptor Expression in Carotid Plaques Associated With Adverse Clinical Events

**Figure 5:**
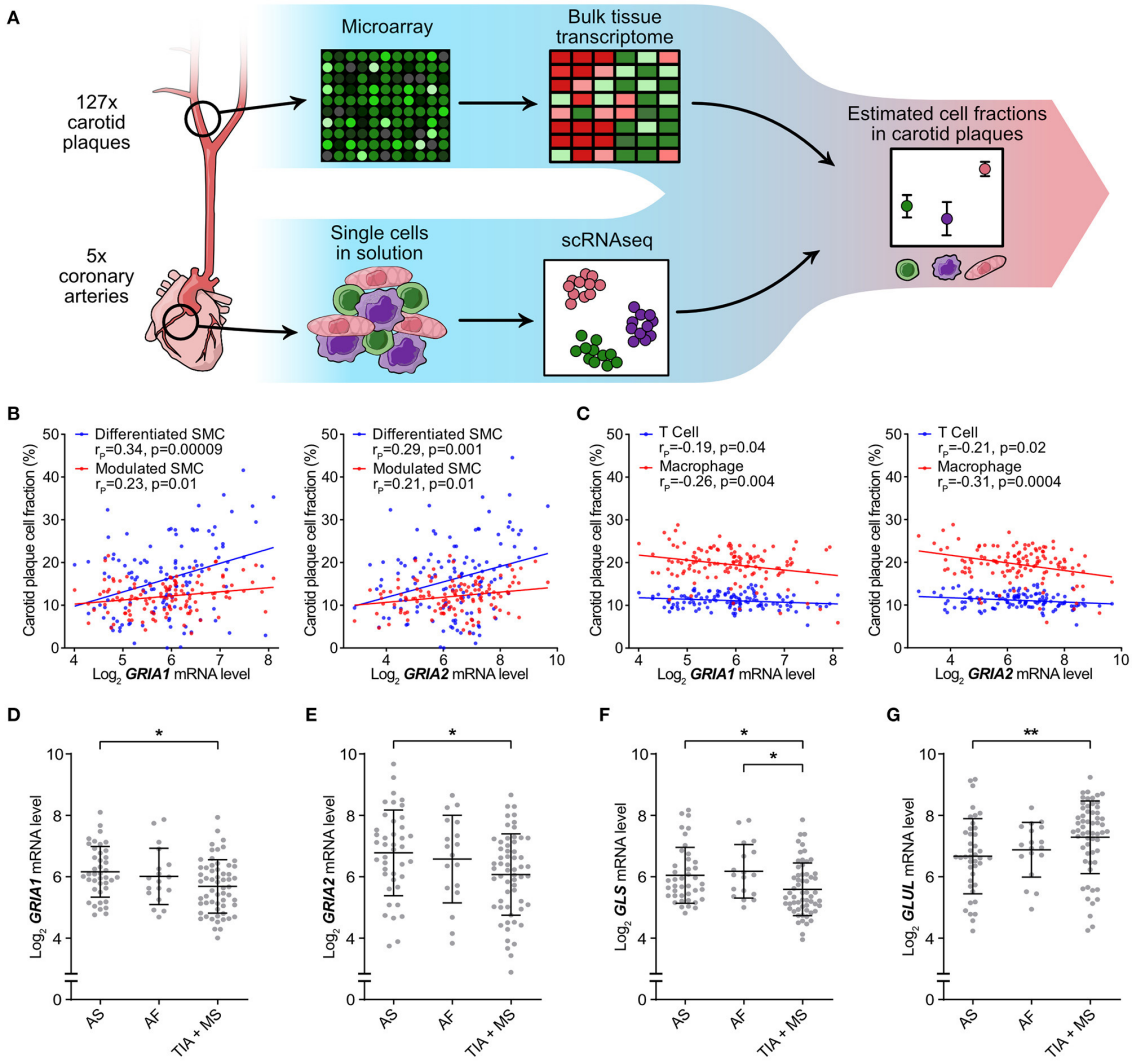
Low AMPA-type Receptor Expression in Carotid Plaques Associated with Adverse Clinical Events. Atheoscelerotic plaque cellular composition in the BiKE cohort was estimated based on microarray data analysis using predefined cellular signatures in atherosclerotic plaques from scRNAseq data. **(A)** Schematic representation of the microarray data analysis process. **(B)** Correlation between *GRIA1* (left) and *GRIA2* (right) mRNA levels and fraction of differentiated (blue) or modulated (red) SMC subtypes in carotid atherosclerotic plaques (r_P_ = Pearson r). **(C)** Correlation between *GRIA1* (left) and *GRIA2* (right) mRNA levels and fraction of T cells (blue) and macrophages (red) in carotid atherosclerotic plaques. (r_P_ = Pearson r). **(D–G)** Dot plots comparing *GRIA1* **(D)**, *GRIA2* **(E)**, *GLS* **(F)**, and *GLUL* **(G)** mRNA levels between asymptomatic (AS) (*n* = 40), *amaurosis fugax* (AF) (*n* = 18), and transient ischemic attack + minor stroke (TIA + MS) (*n* = 59) patients. Dots represent the mRNA level for individual patients. The middle bars indicate mean expression and error bars show SD. Differences between groups were analyzed using Kruskal-Wallis ANOVA followed by Dunn's test. *p < 0.05, **p < 0.01.

Based on the *in vitro* findings of AMPA-type glutamate receptors involvement in VSMC phenotypic modulation, we next investigated whether higher levels of AMPA-type receptor mRNA correlated to increased VSMC content in biopsies from human carotid atherosclerotic plaques. To estimate the cellular composition of carotid plaque biopsies in the BiKE cohort, we performed *in silico* deconvolution of the available whole genome expression data as previously described (23) and based the definitions of cell types on the data from five coronary artery biopsies included in the scRNAseq (22) (Figures 2, 5A). The analysis yielded an estimate of the relative cell population size for the defined cell types in each analyzed plaque (*n* = 127). The relationship between *GRIA1* and *GRIA2* expression, respectively, and the relative abundance of cell populations was analyzed using Pearson correlation (Supplementary Table 6, Figures 5B,C). *GRIA1* and *GRIA2* mRNA levels correlated positively with the inferred relative abundance of VSMCs (Figure 5B) and negatively with the inferred abundance of T cells and macrophages in plaques (Figure 5C), further supporting an association between AMPA-type glutamate receptors and VSMCs in atherosclerosis. Pathway analysis showed that *GRIA1* and *GRIA2* levels in the BiKE plaque microarray data correlated with levels of transcripts associated with VSMC phenotype shift (Supplementary Figure 7).

Next, we investigated whether *GRIA1* and *GRIA2* mRNA levels were associated with severity of adverse clinical events. Patients were stratified in three groups based on the severity of clinical presentation at the time of surgical removal of the carotid lesion, and levels of *GRIA1, GRIA2, GLS*, and *GLUL* compared among the three patient groups. Levels of *GRIA1, GRIA2*, and *GLS* were significantly lower, while *GLUL* was significantly higher, in the group with the most severe symptoms (Figures 5D–G).

### Analysis of Nervous System-Associated Transcripts in Carotid Atherosclerosis Segregates Glutaminase, Glutamate-Ammonia Ligase, and AMPA-Type Receptors

**Figure 6:**
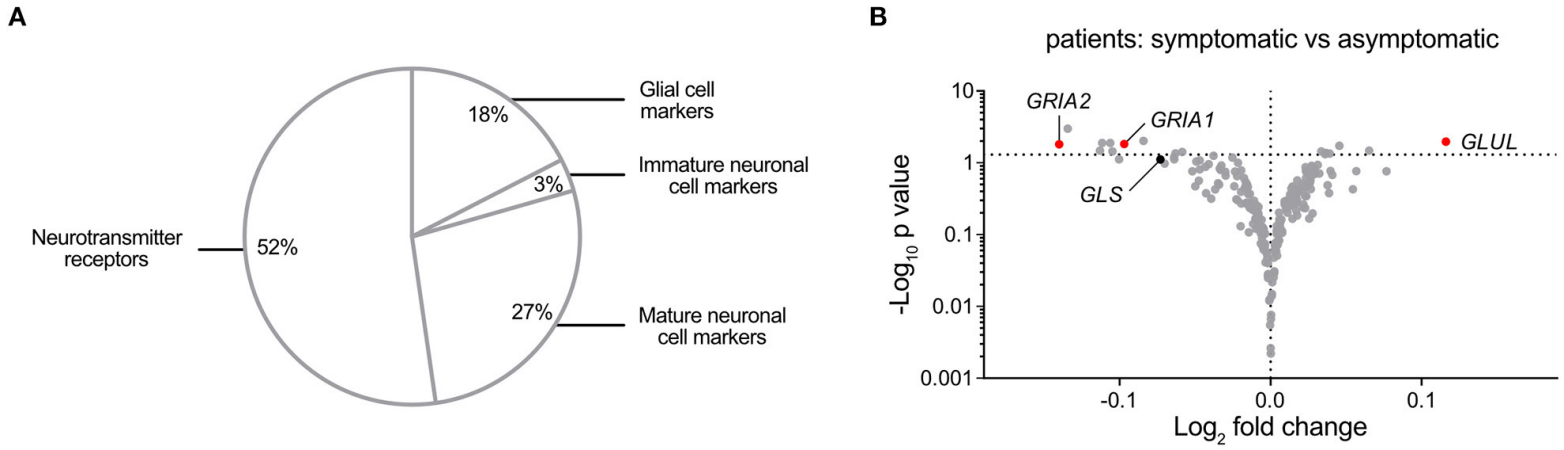
A Comprehensive Analysis of nervous system-associated Transcripts in Carotid Atherosclerosis Segregates Glutaminase, Glutamate-Ammonia Ligase, and AMPA-type Receptors. **(A)** Pie chart showing classification of the compiled 217 neuronal-associated genes investigated by expression analysis in BiKE plaques. **(B)** Volcano plot visualization of differentially expressed genes between symptomatic (*n* = 87) and asymptomatic (*n* = 40) patient groups. Glutamate-related genes are shown in red when their expression is statistically significantly different between conditions, otherwise glutamate-related markers are represented in black. *GRIA1*, Glutamate Ionotropic Receptor AMPA-type Subunit 1; *GRIA2*, Glutamate Ionotropic Receptor AMPA-type Subunit 2; *GLS*, Glutaminase; *GLUL*, Glutamate-ammonia ligase. Fold change is expressed as “log_2_ (mean expression level symptomatic/asymptomatic).” Differences between groups were analyzed using unpaired Student's *t*-test.

Despite reports of neurotransmitter receptor involvement in the pathophysiology of arterial remodeling and atherosclerosis (33, 34), a comprehensive investigation of nervous system-associated transcripts in human atherosclerosis has been lacking. We compiled a list of 217 nervous system-associated components from the literature (35–38) (Supplementary Table 1). The included transcripts were classified by function or cell type association (Figure 6A). Analysis of Affymetrix gene array data from atherosclerotic plaques (*n* = 127) and reference arteries (*n* = 10) in the BiKE cohort were adjusted for multiple comparisons using the Bonferroni-Dunn method and showed significantly different expression of 77 genes between atherosclerotic plaques and reference arteries (Supplementary Table 1). Comparison of mRNA levels of nervous system-associated transcripts between atherosclerotic plaques derived from asymptomatic (*n* = 41) and symptomatic (*n* = 86) patients identified 20 transcripts with significantly different mRNA levels, most prominently transcripts associated with glutamine signaling. In particular, *GLUL* was the most upregulated gene in symptomatic patients, while *GRIA1* and *GRIA2* were significantly downregulated in this group, as compared with the asymptomatic patients group (Figure 6B).

## Discussion

Here we show that AMPA-type glutamate receptors are present in atherosclerotic plaques and associated with phenotypic modulation of VSMCs. Human and mouse atherosclerotic lesion VSMCs express AMPA-type glutamate receptors and pharmacological blocking of these receptors *in vitro* reduced expression of transcripts associated with VSMC phenotypic shift. Atherosclerotic lesions from patients suffering adverse clinical events have significantly lower expression of *GRIA1* and *GRIA2* AMPA-type receptor subunits, compared to those from asymptomatic patients. This suggests that glutamate-signaling might promote contractile VSMC features.

The ubiquitous expression of the glutamate synthesis- and turnover-related enzymes *GLS* and *GLUL* in human atherosclerotic lesions observed here and the reported widespread availability of glutamate (9) supports the notion that the ligand glutamate is available for signaling in human atherosclerotic lesions. The findings suggest that glutamate synthesis may be reduced in atherosclerotic plaques, decreasing the availability of AMPA-receptor ligands, although this was not specifically investigated here. The simultaneous expression of AMPA-type glutamate receptor subunits implies that glutamatergic signaling occurs inside atherosclerotic lesions. Both *GLS* and *GLUL* as well as the glutamate receptor subunits *GRIA1* and *GRIA2* were also among the most differentially expressed genes in carotid atherosclerotic lesions of symptomatic as compared to asymptomatic patients in the BiKE cohort. In light of these observations, it is interesting to consider whether glutamate signaling may be an active component that regulates pathophysiology of atherosclerotic lesions. Considering that lower receptor levels were found in patients with more severe symptoms, it will be important to study whether increased AMPA-receptor activation by glutamate has a protective effect in atherosclerosis.

Glutamate receptors *GRIA1* and *GRIA2* were essentially restricted to plaque VSMCs both in the coronary artery and in carotid artery atherosclerotic lesions. The published observations that phenotypic modulation is associated with glutamate signaling in VSMC (2) supports the hypothesis that AMPA-type glutamate receptor levels are linked with VSMC phenotype. In line with this, we observed a significant induction of *Gria1* expression in carotid arteries undergoing vascular remodeling *in vivo*, particularly at times after injury when VSMC activation toward intimal invasion is known to be pronounced (24). After arterial injury, contractile VSMCs respond with a phenotypic switch, turning into modulated VSMCs, which are characterized by high proliferative and migratory activity (2). In this experimental model, modulated VSMCs increasingly populate the intimal surface between days 2 and 5 (24). These observations on the glutamate signaling machinery in arterial repair are noteworthy and may have consequences also in atheroprogression given the important role of VSMCs in the vessel wall, promoting plaque stability and encapsulating plaque inflammation through formation of the fibrous cap, likely promoting an atheroprotective phenotype (2, 3, 8).

In addition to its role as a ligand for glutamate receptors, glutamate is a vital metabolite that links carbon and nitrogen metabolism (39) and is also an important constituent of growth media for VSMCs. It is therefore reasonable to assume that ligand availability is plentiful for glutamate receptors expressed by VSMCs both *in vivo* and *in vitro*, and functional studies should thus be preferably performed using selective glutamate receptor blockers. The limitation of using NBQX is that it is a selective AMPA-type receptor antagonist with antagonist effects also on Kainate-type receptors. However, NBQX is a well-characterized and reportedly a non-toxic antagonist which has been abundantly used in *in vivo* studies for its capacity of preventing glutamate-dependent CNS damage (26). In the hcSMC studied here, NBQX did not significantly affect apoptosis. As could be expected from the *in vivo* and *in vitro* observations by Dumas et al. (14), abrogating glutamate signaling by NBQX reduced proliferation also in hcSMCs. Our observations that blocking AMPA-type glutamate receptors such as GRIA1 in cultures of hcSMCs triggered significant regulation of a set of genes associated with extracellular matrix organization, cell adhesion, wound healing and regulation of protein binding, indicate that activity of glutamate receptors in VSMCs is involved in processes that are drivers of VSMC activity and phenotype. Several markers associated with differentiated VSMCs, including SRF (a key transcription factor in VSMC biology), were significantly lower in the presence of NBQX. Interestingly, a known early key transcription factor in differentiation toward the VSMC lineage, MYOCD, was significantly higher in samples exposed to NBQX. While these observations make it tempting to speculate on glutamate signaling involvement in VSMC differentiation, it is difficult to draw firm conclusions based on these data, not least since blocking of AMPA-receptor changed SRF and MYOCD mRNA levels in opposite directions. Additionally, numerous transcripts that were regulated by pharmacological blocking of AMPA-type glutamate receptors in hcSMC *in vitro* have been previously linked to plaque stability. For example, it has been reported that retinoic acid (RA) reduces plaque formation in an atherosclerosis prone mouse model (40), and here, we observed an upregulation of RA receptors (*RARB*) and two RA receptor responsive genes (*DHRS3* and *RARRES1*) (41) following abrogation of AMPA receptor signaling. Another example is hyaluronic acid (HA), which is a component of the extracellular matrix synthetized by *HAS2*. HA plays diverse roles, depending on the concentration and on the molecular size (42), in plaque formation. We observed that *HAS2* is downregulated in the presence of an AMPA receptor antagonist. In light of these observations, regulation of these genes and perhaps VSMC phenotype itself (2) in a beneficial direction in atherosclerosis might be worth evaluating further using drugs that promote glutamatergic signaling.

In patients with more severe ischemic clinical events, grouped as a transient ischemic attack (TIA) and minor stroke (MS), the mean expression level of AMPA-type glutamate receptors *GRIA1* and *GRIA2* was lower than in individuals with less severe adverse vascular symptoms (*amaurosis fugax*, AF) or without reported symptoms. There was also a significant correlation between plaque *GRIA1* mRNA levels and plasma IL-6, a pro-atherogenic cytokine (43). Selective pharmacological blocking of AMPA-type glutamate receptors in hcSMCs *in vitro* promoted a significant increase of *IL-6* mRNA levels and secretion of IL-6 to the extracellular space. Although we were unable to measure plaque IL-6 protein levels and perform selective blocking of AMPA-type glutamate receptors *in vivo*, our observations suggest that plaque glutamate receptors may reduce inflammation and further mechanistic investigation would be of interest.

Carotid VSMCs and several neuronal cell types share a common embryonic origin, the neural crest (44). It is conceivable that glutamate signaling may not be the only neurotransmitter involved in VSMC differentiation and, by extension, the pathophysiology of atherosclerosis. The neurotransmitter receptor cholinergic alpha 7 nicotinic acetylcholine receptor subunit (α7nAChR) is expressed in human atherosclerotic plaques and experimental observations support that receptor activation by the neurotransmitter acetylcholine (ACh) attenuates atherosclerosis progression (34, 45). Furthermore, arteries are innervated by adrenergic nerves, and efferent neural signals can regulate vascular contraction (46). It has been suggested that innervation of the vascular adventitia can be involved in neuro-immune crosstalk between layers of the vascular wall (47). Despite the reports on neurotransmitter signaling in vascular biology and atherosclerosis, a comprehensive map of nervous system-associated signaling components expressed in atherosclerotic plaques has been lacking. The analysis of nervous system-associated transcripts in human atherosclerosis presented here revealed significant differences in transcript levels both between reference arteries and atherosclerotic plaques and between patients classified as symptomatic and asymptomatic in the BiKE cohort. Neurotransmitter signaling is hitherto rather unexplored in atherosclerosis, and mapping of neural control of inflammation in other conditions with chronic inflammation has opened for new therapeutic opportunities and clinical trials of anti-inflammatory effects of specific nerve activation (48–51). Although some observations suggest that peripheral nerve activity regulate atherosclerosis inflammation (52), atherosclerotic lesions are not known to be directly innervated and the potential function of neural regulation of inflammation in atherosclerotic lesions remains to be mechanistically explored. The analysis of nervous system-associated transcripts in human atherosclerotic lesions described here may provide a starting point for further exploration and discovery of key nervous-system related signaling in atherosclerosis.

In conclusion, our results from transcriptomic microarray analysis and single cell RNA sequencing show that AMPA-type glutamate receptors are expressed by VSMCs both in arterial repair and in atherosclerotic plaques, and at lower levels in plaques from patients that suffered adverse clinical events. Blocking AMPA-type glutamate receptors in human carotid artery VSMCs promoted expression of gene sets associated with VSMC phenotypic modulation, providing evidence of functional glutamate signaling in human VSMCs. Moreover, components of the glutamate signaling axis were identified as the most differentially expressed nervous system-associated genes in plaques from asymptomatic compared with symptomatic patients. These observations support the notion that neurotransmitter signaling may play yet unexplored roles in vascular biology and further mechanistic studies of neural regulation of atherosclerosis pathogenesis are warranted.

## Data Availability Statement

The datasets presented in this study can be found in online repositories. The names of the repository/repositories and accession number(s) can be found at: European nucleotide archive, accession numbers PRJEB43287, ERP127239.

## Ethics Statement

The BIKE study was approved by the Ethical Committee of Stockholm and was performed in agreement with institutional guidelines and the principles of the Declaration of Helsinki. The following ethical permits apply: BiKE EPN DNr 95-276/277; 01-199; 02-146; 02-147; 04-225/4; 04-97 5T; 2005/83-31; 2007/281-31/4; 2009/4:2; 2009/9-31/4; 2009/295- 31/2; 2009/512-31/2; 2009/2000-32; 2010/1022-31/1; 2010/730-31/2; 2011/196-31/1; 2011/629-32; 2011/950-32; 2012/619-32; 2012/916-31/4;2012/1096-31/2; 2012/1279-32; 2013/615-31/4; 2012/2188-31-5; 2013/2048-32; 2013/2137-32; 2015/1338-32; 2015/2108-31/5; 2017/508-32; and 2018/954-32. Studies involving human coronary arteries were approved by the Stanford University Institutional Review Board. Informed consent was provided for all enrolled by themselves or their legal guardian. Animal studies were approved by the Administrative Panel on Laboratory Animal Care at Stanford University and the Ethical Board of North Stockholm, Dnr N181/16; N137/14, respectively, and conform to Institutional Guidelines.

## Author Contributions

AGa, PO, and LM conceived the study, planned experiments, analyzed, and interpreted data. AGa, LT, AC, PO, and LM wrote the manuscript. AGa, UR, RW, AC, SM, VS, MY, GK, ML, and AR performed experiments and analyzed data. AGi, GP-B, TQ, SM, and VS edited the manuscript. All authors contributed to the article and approved the submitted version.

## Conflict of Interest

PO is a co-founder of and shareholder in ChAT Therapeutics and Emune AB. The remaining authors declare that the research was conducted in the absence of any commercial or financial relationships that could be construed as a potential conflict of interest.
