# Supplementary material for: AMPA-Type Glutamate Receptors Associated With Vascular Smooth Muscle Cell Subpopulations in Atherosclerosis and Vascular Injury
Source: Front Cardiovasc Med. 2021 Apr 20;8:655869. doi: 10.3389/fcvm.2021.655869 (PMC8093397; doi:10.3389/fcvm.2021.655869)
Supplement: Supplementary file 1 [file Data_Sheet_1.docx]

Supplementary Material


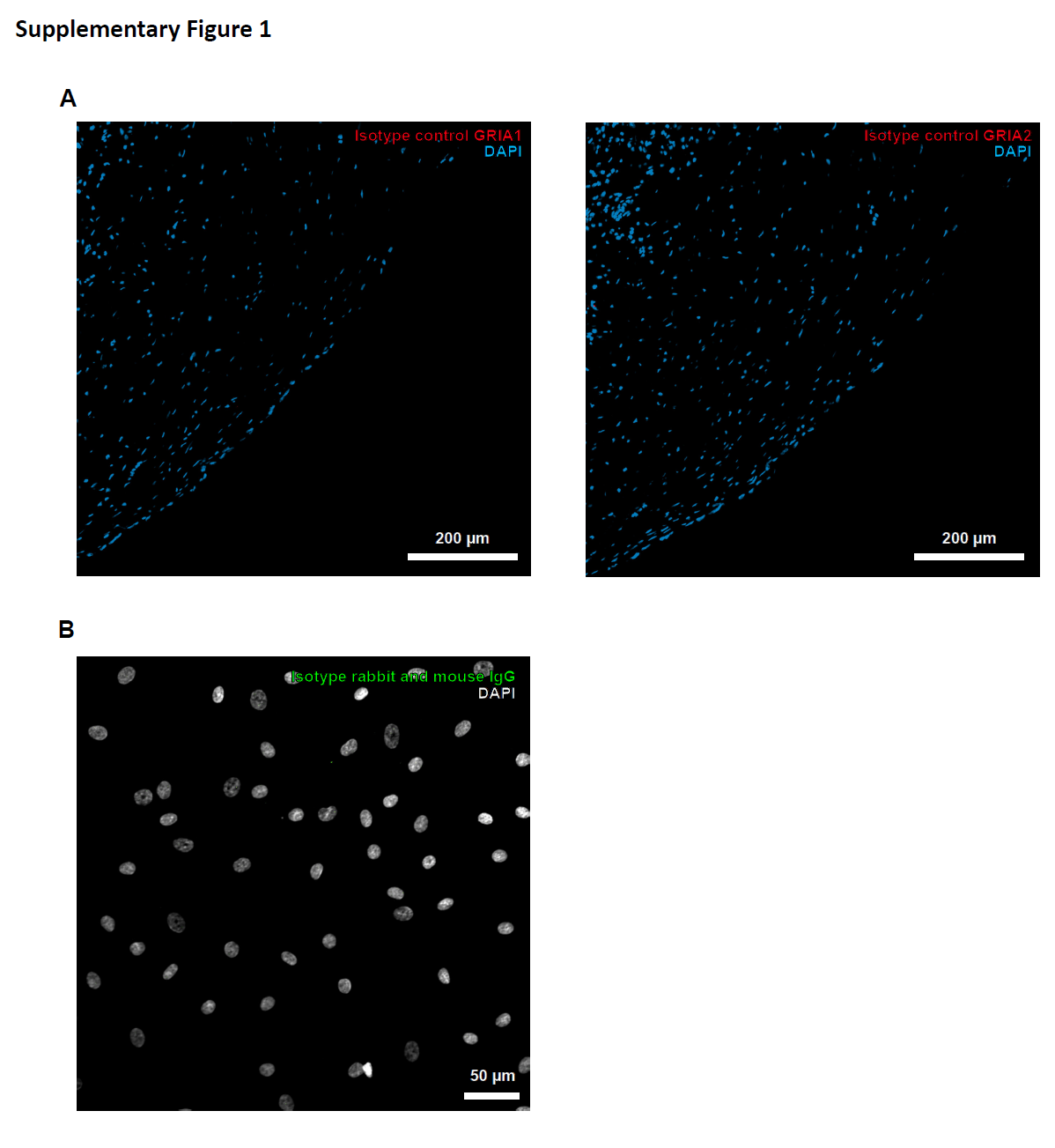


**Supplementary Figure 1**: (A) Consecutive sections from the representative human carotid plaque from BiKE cohort shown in Figure 1E and 1F were stained using corresponding isotype control antibody for GRIA1 (left) and GRIA2 (right), both followed by a biotinylated anti-rabbit secondary antibody and streptavidin (red). Nuclei were stained with DAPI (blue).

(B) Negative control for Figure 4B, C. human carotid SMCs maintained in serum free media for 24 h were stained using an isotype rabbit and mouse IgG followed by a biotinylated anti-rabbit secondary antibody and streptavidin (green), and DAPI (white).


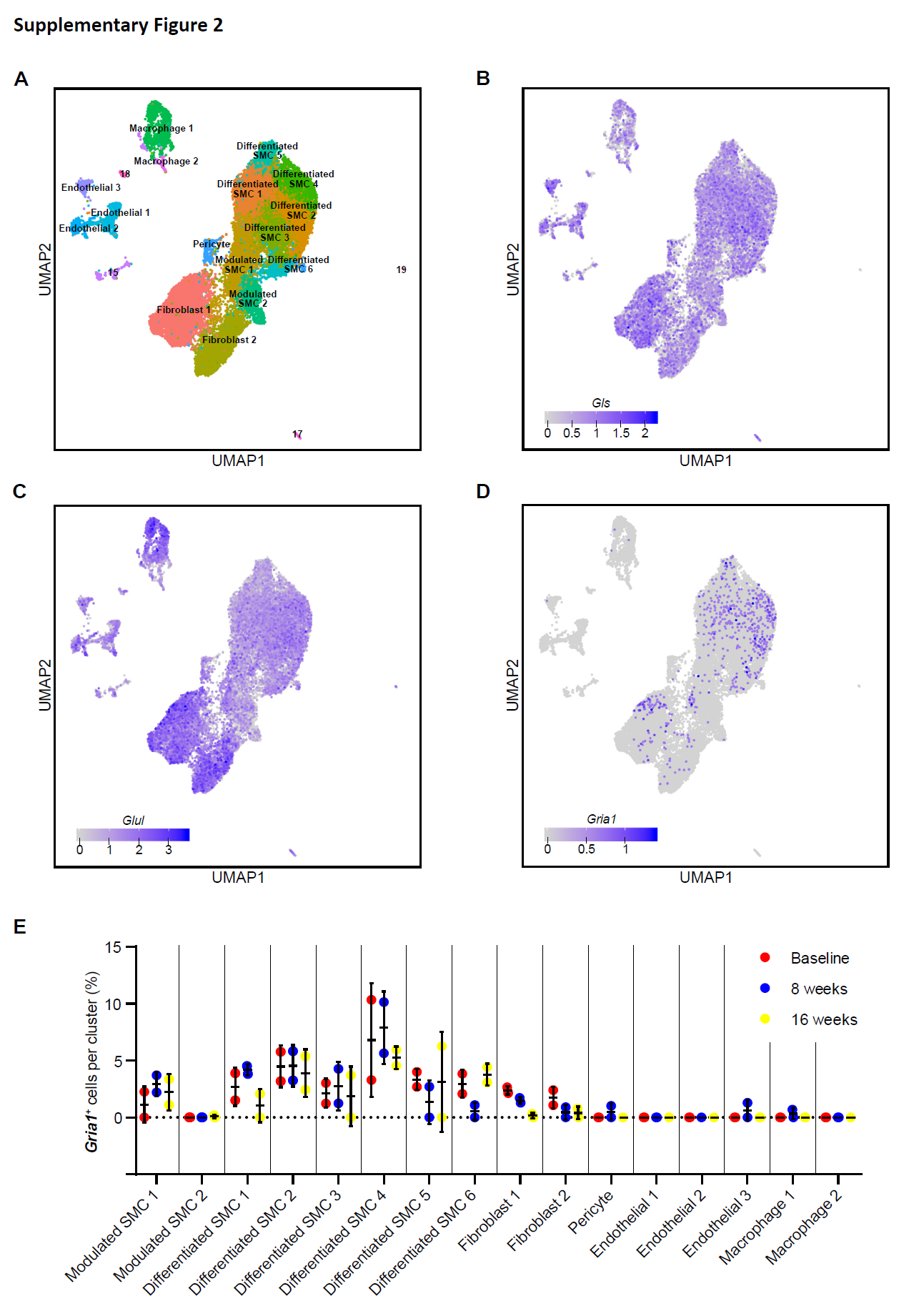


**Supplementary Figure 2**: Single cell RNAseq data from SMC lineage-tracing atherosclerosis-prone mice.

(A) UMAP visualization of single cell transcriptomic analysis of cell types present in the mouse aortic root at baseline (n = 3 mice), after 8 weeks of high fat diet (n = 3 mice) and after 16 weeks of HFD (n = 3 mice). Cell clusters are colored according to cell identity, as indicated in the figure. Numbers denote unidentified clusters. SMC = smooth muscle cell.

(B) UMAP visualization of single cell transcriptomic analysis of *Gls* (blue) expression overlaid on the cell clusters from (A). Color legend indicating relative expression levels for *Gls*.

(C) UMAP visualization of single cell transcriptomic analysis of *Glul* (blue) expression overlaid on the cell clusters from (A). Color legend indicating relative expression levels for *Glul*.

(D) UMAP visualization of single cell transcriptomic analysis of *Gria1* (blue) expression overlaid on the cell clusters from (A). Color legend indicating relative expression levels for *Gria1*.

(E) Dot plot visualization of the percentage of cells positive for *Gria1* expression within the identified cell clusters from mouse aortic root at baseline (red; n=3 mice; n=2 experiments), after 8 weeks of HFD (blue; n=3 mice; n=2 experiments) and after 16 weeks of HFD (yellow; n=3 mice; n=2 experiments). Error bars indicate SD.


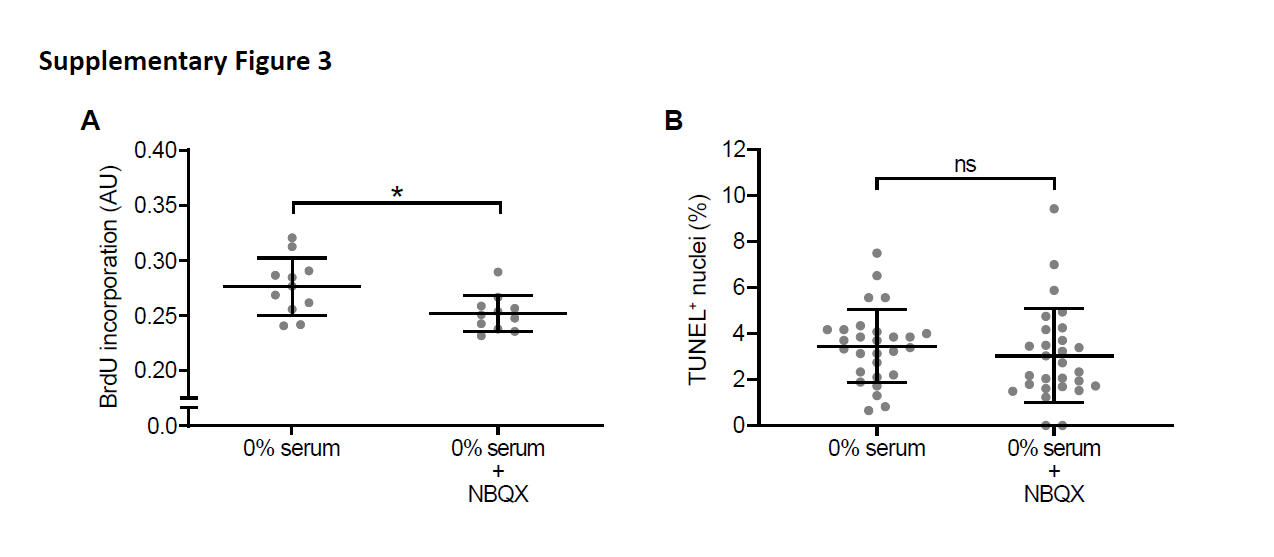


**Supplementary Figure 3**: (A) Dot plot visualization of BrdU incorporation levels in human carotid artery-derived SMCs in vitro exposed to the AMPA-type receptor antagonist NBQX or vehicle (n=11). Middle lines indicate mean with error bars indicating SD. *p<0.05 (unpaired Student’s t-test).

(B) Dot plot visualization of percentage of TUNEL positive nuclei immunofluorescent imaging stained (n=6 pictures for each of the n=4 glass coverslips) in human carotid artery-derived SMCs *in vitro* exposed to the AMPA-type receptor antagonist NBQX or vehicle. Middle lines indicate mean with error bars indicating SD. ns = non-significant (unpaired Student’s t-test).


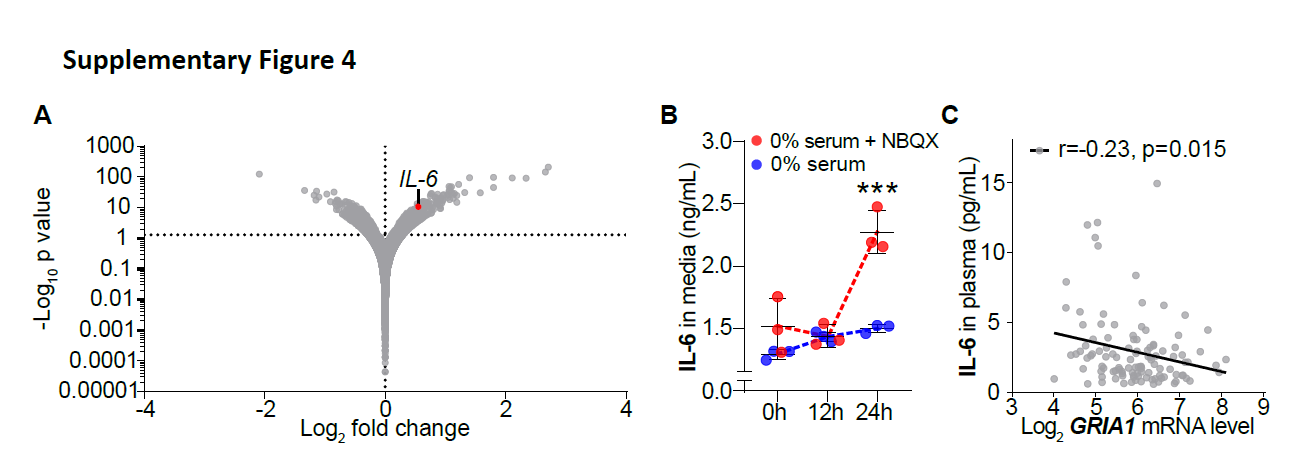


**Supplementary Figure 4**: (A) *Interleukin 6* (*IL6*) gene is highlighted (red) in the volcano plot in Figure 4D, representing fold change and p values of differently expressed genes between hcSMCs grown in 0% serum media in the presence (n=3) or absence (n=3) of the AMPA receptor antagonist NBQX for 24 h.

(B) hcSMC were grown in serum-free media in the presence (red) or absence (blue) of the AMPA receptor antagonist NBQX and IL-6 measured in culture supernatants by ELISA. Lines show mean ± SD. ***p<0.001 (unpaired Student’s *t*-test).

(C) IL-6 was measured in plasma from 112 patients in the BiKE cohort and plotted against *GRIA1* mRNA levels in the corresponding atherosclerotic plaques. (r_P_ = Pearson r).


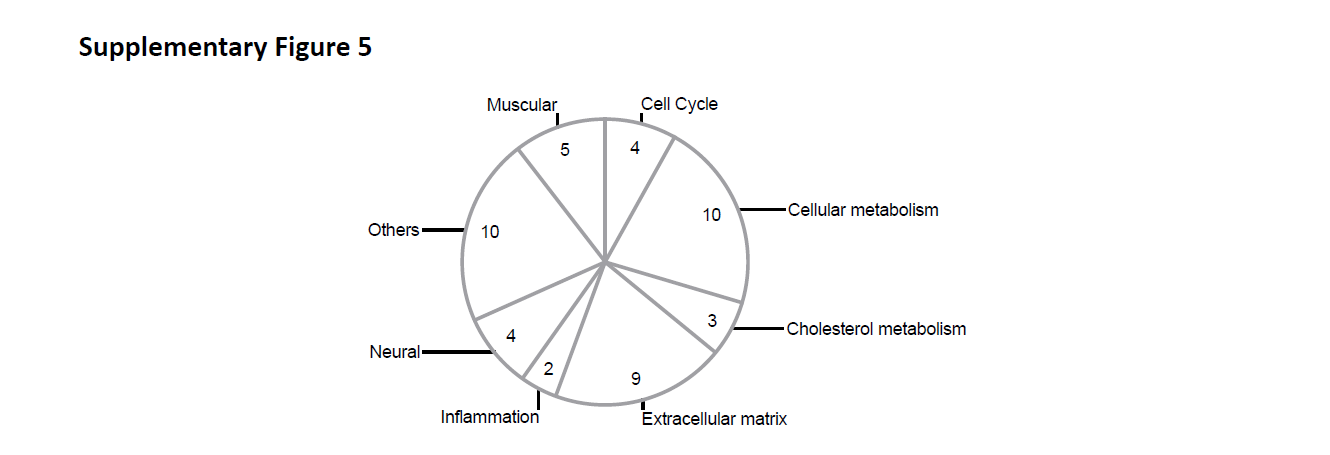


**Supplementary Figure 5**: Pie chart illustrating the 47 mostly affected GO-pathways, filtered by REVIGO software and classified by function, following AMPA receptor inhibition in hcSMCs. Number of pathways for each category is reported inside the chart.


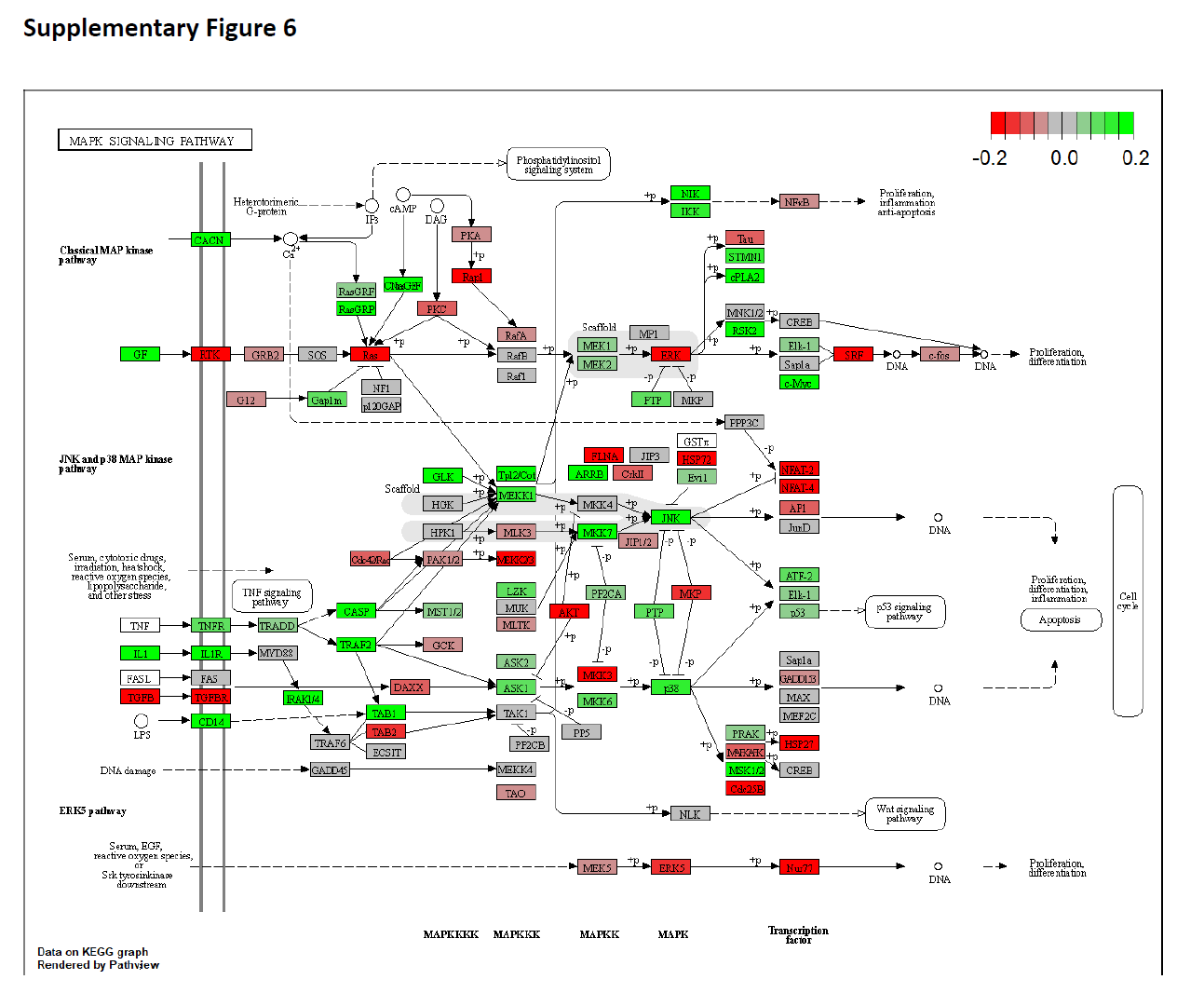


**Supplementary Figure 6**: Pathway analysis of RNAseq data from hcSMCs grown in serum free media in the presence (n=3) or absence (n=3) of the AMPA receptor antagonist 2,3-dihydroxy-6-nitro-7-sulfamoyl-benzo[f]quinoxaline disodium salt (NBQX) for 24 h. TPM were mapped onto MAPK signaling cascade KEGG pathways by R package "Pathview". Genes are described as boxes that have been colored according to expression levels.


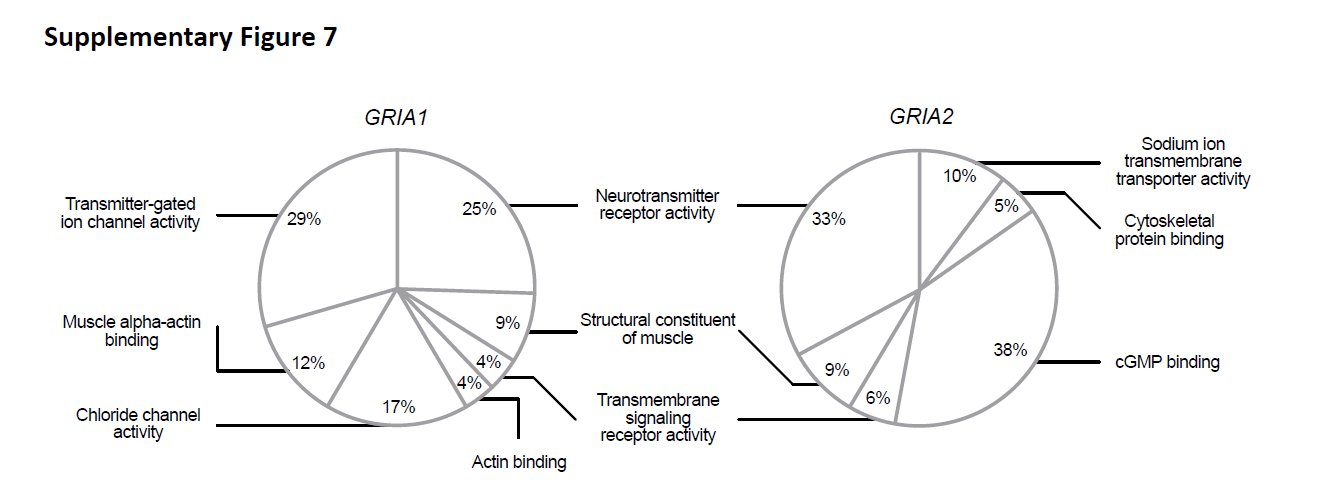


**Supplementary Figure 7**: Pie chart illustrating the gene set enrichment in pathway analysis of global transcriptomic correlations based on *GRIA1* (left) and *GRIA2* (right) expression in BiKE microarray data from human carotid plaques, analyzed using GOrilla software. Percentage of enrichment score is reported inside the charts.

**Supplementary Tables 1-6 are presented in the attached excel file.**

**Supplementary Table 1**: Microarray array expression levels of 217 nervous system-associated transcripts in BiKE cohort in atherosclerotic carotid plaques (n=127) and non-atherosclerotic reference arteries (n=10). Differences between the groups were calculated as fold change as “log_2_ (average carotid atherosclerotic plaque/average non-atherosclerotic arteries)”. Statistical analysis was performed using two-tailed Student’s *t*-test.

**Supplementary Table 2**: Average number of positive cells for defined markers in the 20 defined cell clusters in coronary atherosclerotic plaques (n=4). “Total” represents the average number of cells in the corresponding cluster.

**Supplementary Table 3**: Number of reads in RNAseq analysis for the 464 significantly differently expressed genes between hcSMCs grown in 0% serum media in the presence (n=3) or absence (n=3) of the AMPA receptor antagonist NBQX for 24 h.

**Supplementary Table 4**: Gene ontology classification of the differently expressed genes from RNAseq analysis of hcSMCs grown in 0% serum media in the presence (n=3) or absence (n=3) of the AMPA receptor antagonist NBQX for 24 h.

**Supplementary Table 5**: GO terms describing signaling pathways were filtered according to significance (p < 0.05). Acronym – terms used to query KEGG pathways to visualize genes in gene expression using pathview R/Bioconductor package.

**Supplementary Table 6**: Pearson correlation of *GRIA1* and *GRIA2* mRNA levels in the BiKE cohort and the fraction of cell types in the carotid plaque as estimated by *in silico* deconvolution.
